# PReS-FINAL-2325: Different phenotypes associated with Q703K variant of the NLRP3 gene

**DOI:** 10.1186/1546-0096-11-S2-P315

**Published:** 2013-12-05

**Authors:** A Von Scheven-Gête, M Hofer, G Simon, N Busso, M Morris, F Vanoni

**Affiliations:** 1 CHUV, Lausanne, Switzerland; 2 Synlab, Lausanne, Switzerland

## Introduction

PFAPA syndrome is an auto-inflammatory disease (AID), characterized by recurrent fever aphtosis, pharyngitis and cervical adenitis. A familial predominance has been observed and variants in the NLRP3 gene were recently found in several patients. The NLRP3 Q703K variant was described so far in CAPS patient and in healthy carriers.

## Objectives

To describe the different phenotypes of patients with recurrent fever and Q703K variant

## Methods

In patients presenting to our consultation with recurrent fever suspected to be auto-inflammatory, we screened genomic DNA by PCR and sequenced for genetic variants of NLRP3 genes. The symptoms, treatment, response to treatment and family history of the patients with the variant Q703K have been retrospectively extracted and described.

## Results

We found the NLRP3 variant Q703K in 13 patients. Ten were PFAPA patients among 97 from our cohort; one had a CAPS phenotype and two an undefined autoinflammatory disease (UAID): 9 boys and 4 girls with a median age of 18 months at disease onset. Family history was positive in 6 PFAPA and one UAID patients. For PFAPA, the median duration of fever was 4 days; the median interval was 4 weeks. Pharyngitis and cervical adenitis was always present in 6 patients. Aphtosis was found only in 1 patient in every episode. 5 patients expressed abdominal pain that accompanied most fever episodes. 1 patient showed sometimes arthralgia, 2 patients had headaches in most episodes and one patient had once a cutaneous rash. 5 out of 7 patients treated by corticosteroids responded promptly. In the other two patients two doses were often necessary. 3 patients underwent tonsillectomy: one with no effect, in 2 the fever episodes resolved but one patient had persistent episodes of aphtosis. In 4 patients genomic sequencing of the parents was done; one parent positive for Q703K had a history of recurrent febrile episodes, but the 3 other parents did not present a history of recurrent fever episodes nor recurrent pharyngitis nor tonsillectomy. The patient with CAPS phenotype presented with urticarial rash, partial deafness, arthralgias and elevated inflammatory parameters, and responded well to IL-1 blocking agents. One patient with UAID presented recurrent fever episodes with neurological symptoms (hypotonia, bulging fontanelle, loss of contact) and high inflammatory markers, and the second with fever flares and angioedema. In one UAID and two PFAPA patients another heterozygous variant in the MEFV gene was found.

## Conclusion

We report 13 patients positive for the NLRP3 Q703K variant and presenting different phenotypes, mainly PFAPA syndrome. The role of this variant in the clinical manifestations of our patients is challenged by the negative history for fever syndromes in the Q703K+ PFAPA parents. The presence of a second variant in a gene involved in recurrent fever syndromes in 3 of our 13 patients suggests that another gain-of-function mutation is necessary in patients with the Q703K variant to induce auto-inflammatory manifestations.

## Disclosure of interest

None declared.

